# The Texas State Medical Association; Reorganization; Revision of Constitution

**Published:** 1901-09

**Authors:** 


THE TEXAS STATE MEDICAL ASSOCIATION; REORGANIZATION; REVISION OF CONSTITUTION. A committee has been appointed to revise the constitution and by-laws of the Texas State Medical Association and make it conform with the changes recently adopted by the American Medical Association. Dr. West, secretary of the State Medical Association, in a circular letter to the secretaries of the various local societies thus outlines the proposed changes:

First: Admission of all members of affiliating societies as members of the State Association. Second: Reduction of annual dues, probably to $3 per annum. Third: The government of the State Association to be in the hands of a House of Delegates, appointed by affiliating societies. Fourth: The adoption of a comprehensive plan of reorganization of county and district societies.

Briefly stated, the most important of the changes recommended by the National Association's committee on reorganization is as follows:

The creation of a delegate body to be known as the "House of Delegates," not to exceed one hundred and fifty in number, and to be made up as follows: (a) one delegate for every five hundred members, or fraction thereof, of the State or Territorial societies;

(b) one delegate from each of the thirteen sections of the American Medical Association; and (c) one representative each from the army, navy and marine hospital service. This body shall constitute the Section on Business, and shall hold its meetings daily during the session of the Association.

The advantages to be thus gained are readily apparent:

First: The business and scientific work will be separated. Second: All business, much of which has heretofore been neglected, will now receive due attention and be properly disposed of by a small, well selected and representative body. Third: Every State will be fairly represented, and States near the place of meeting cannot, hereafter, exert a controlling influence in the meetings.

With reference to the reoganization of the subordinate societies, the committee points out that no successful outcome is possible without the cordial co-operation of the State and National societies, and that a common plan of organization must be adopted by all.

The real objects of the State Medical Association are thus stated:

"The whole duty of a State society consists in doing all in its power to better the conditions of every individual member of the medical profession in its territory. A body whose sole aim and object is to benefit only those who attend its annual meetings should cease to exist, or else change its name and claims, so that a State society could be organized that would appreciate its full duty and do it. * * * The State society, representing the profession of the State, should have cognizance of all medico-political, social and financial measures affecting the profession, as well as sanitary affairs that affect the well-being of the people. It should be ready at all times to oppose measures and undertakings, whether originating in or out of the profession, that have a tendency to degrade it and lower its standard as a scientific body of men, or that would affect the profession disadvantageously in any way."

"In addition to the above, but not more important, is its function as the great scientific medical body of the State."

"Hence, as in the organization of the American Medical Association, there should be two distinct branches: the scientific and legislative."

The preliminary report of the committee is contained in the May 25 number of the *Journal of the American Medical Association*, and is well worth a careful reading by every physician who is interested in the welfare of the profession. W. B. R.

